# Mutations in *RABE1C* suppress the *spirrig* mutant phenotype

**DOI:** 10.1371/journal.pone.0304001

**Published:** 2024-06-17

**Authors:** Marc Jakoby, Lisa Stephan, Björn Heinemann, Martin Hülskamp

**Affiliations:** 1 Botanical Institute, Biocenter, Cologne University, Cologne, Germany; 2 Institute for Plant Sciences, Cluster of Excellence on Plant Sciences (CEPLAS), Cologne University, Cologne, Germany; DREAM Tech LLC, UNITED STATES

## Abstract

The plant BEACH-domain protein SPIRRIG (SPI) is involved in regulating cell morphogenesis and salt stress responses in *Arabidopsis thaliana*, *Arabis alpina*, and *Marchantia polymorpha* and was reported to function in the context of two unrelated cellular processes: vesicular trafficking and P-body mediated RNA metabolism. To further explore the molecular function of SPI, we isolated a second-site mutant, specifically rescuing the *spi* mutant trichome phenotype. The molecular analysis of the corresponding gene revealed a dominant negative mutation in *RABE1C*, a ras-related small GTP-binding protein that localizes to Golgi. Taken together, our data identified the genetic interaction between RABE1C and SPI, which is beneficial for further dissecting the function of SPI in vesicle trafficking-associated cell morphogenesis.

## Introduction

BEACH domain-containing proteins (BDCPs) are well-conserved in mammals, plants, and yeast and were initially found to be involved in membrane dynamics and endosomal sorting processes [1, 2]. The best described BDCP in plants is the *SPIRRIG* (*SPI*) gene [3–8].

In the N-terminus, SPI carries tandem Armadillo repeats with a conserved three-dimensional structure of three α helices, which can fold together and interact to form a surface for protein-protein interactions [9]. The Armadillo repeats are followed by a Concanavalin A (ConA)-like lectin domain, which is thought to be involved in oligosaccharide binding that mediates membrane fusion events [10]. In the C-terminus, SPI exhibits a Pleckstrin-Homology (PH) domain, followed by the name-giving BEACH domain. PH domains were found to interact with BEACH domains to form a large groove, possibly serving as a ligand-binding site [11]. Like in most BDCPs, the BEACH domain of SPI is followed by several WD40 repeats, which can mediate protein-protein interactions [5]. Morphological *spi* phenotypes in *Arabidopsis thaliana* include weakly distorted and curled trichomes, low-complexity epidermal pavement cells, and stunted root hairs [5]. In 2021, Chin et al. studied the mechanistic role of SPI in root hair morphogenesis in great detail: Root hair expansion occurs only at the very tip of the cell and depends on tip-localized actin. SPI co-localizes with actin at the tip of growing root hairs and is vital for actin localization. SPI regulates actin possibly through BRICK1, which regulates actin organization through the ARP2/3 complex. BRICK1 usually is removed from the incipient tip and absent in the tip during root hair elongation. In *spi* mutants, BRICK1 remains at the tip. It is unknown whether SPI destabilizes BRICK1 or is involved in the transport dynamics of BRICK1 at the membrane. In *A*. *thaliana*, its close Brassicaceae relative *Arabis alpina*, and one of the first land plants, *Marchantia polymorpha*, SPI has been found to act in two seemingly unrelated molecular pathways apart from its connection to BRICK1. First, SPI functions in endosomal trafficking. This is suggested by the physical interaction of SPI with and localization to ATPase Suppressor of K+-Transport Growth Defect 1 (SKD1) and LYST Interacting Protein 5 (LIP5) [3, 6, 7]. Also, in root hairs of *A*. *thaliana*, defects in vacuolar integrity were described [5]. Second, SPI is involved in mRNA metabolism. In support of this, SPI is localized to and facilitates the formation of mRNA processing bodies (P-bodies) [3, 7, 8]. These similarities indicate that many aspects of SPI function are evolutionarily conserved, and indeed, *A*. *alpina spi* mutants show a similar spectrum of morphological phenotypes to *A*. *thaliana* [7]. In *M*. *polymorpha*, a short rhizoid phenotype was found in *spi* mutants [3], and salt hypersensitivity is characteristic for *spi* mutants in all three species [3, 7, 8].

One possibility to explore the molecular function of SPI further is the search for genetic modifiers. Genetic modifiers are mutations in a second gene that affect the phenotype of the analyzed mutant and are, therefore, functionally linked. In this work, we took this approach and identified a second-site mutation rescuing the *spi* mutant phenotype. The molecular analysis revealed that the corresponding gene encodes *RABE1C*, a ras-related small GTP-binding protein. Small GTP-binding proteins act as molecular switches activated by GTP and deactivated through hydrolysis of GTP to GDP. Guanine nucleotide exchange factors (GEFs) control these switches by catalyzing the conversion to their active state. In this active state, they interact with downstream effector proteins for diverse cellular functions. Deactivation can occur through intrinsic GTP hydrolysis or binding to GTPase-activating proteins (GAPs), returning them to an inactive state [12, 13]. Rab GTPases, which are part of the ras superfamily, are crucial for targeting specificity in eukaryotic membrane traffic [14–16]. They regulate tethering factors and possibly SNARE complexes in yeast and mammalian cells, facilitating vesicle and organelle membrane docking and fusion. Some Rab GTPases also enhance transport vesicle-cytoskeleton interactions [15, 16]. Various Rab GTPase members are responsible for distinct vesicle-targeting events. They interact with regulatory and effector molecules, linking GTP-binding and GTP-hydrolysis to vesicle processes [15, 16]. Arabidopsis encodes 57 Rab GTPases [12] grouped into eight clades [17] with 18 structural subclasses [18]. The RabE subclass is related to post-Golgi Rab subclasses and is homologous to RAB8 and RAB10 in mammals, which are known or suspected to be involved in post-Golgi transport to the plasma membrane [17].

## Results

### 1.1 Identification and phenotypic characterization of a suppressor of *spi*

To explore the molecular function of SPI, we performed a screen for mutants modifying the *spi* mutant phenotype. Towards this end, we mutagenized the T-DNA allele *spi-4* (Col-0 background) with EMS and screened the M2 generation for plants displaying trichome rescue. In this work, we studied the line M20 in more detail.

**Fig 1:**
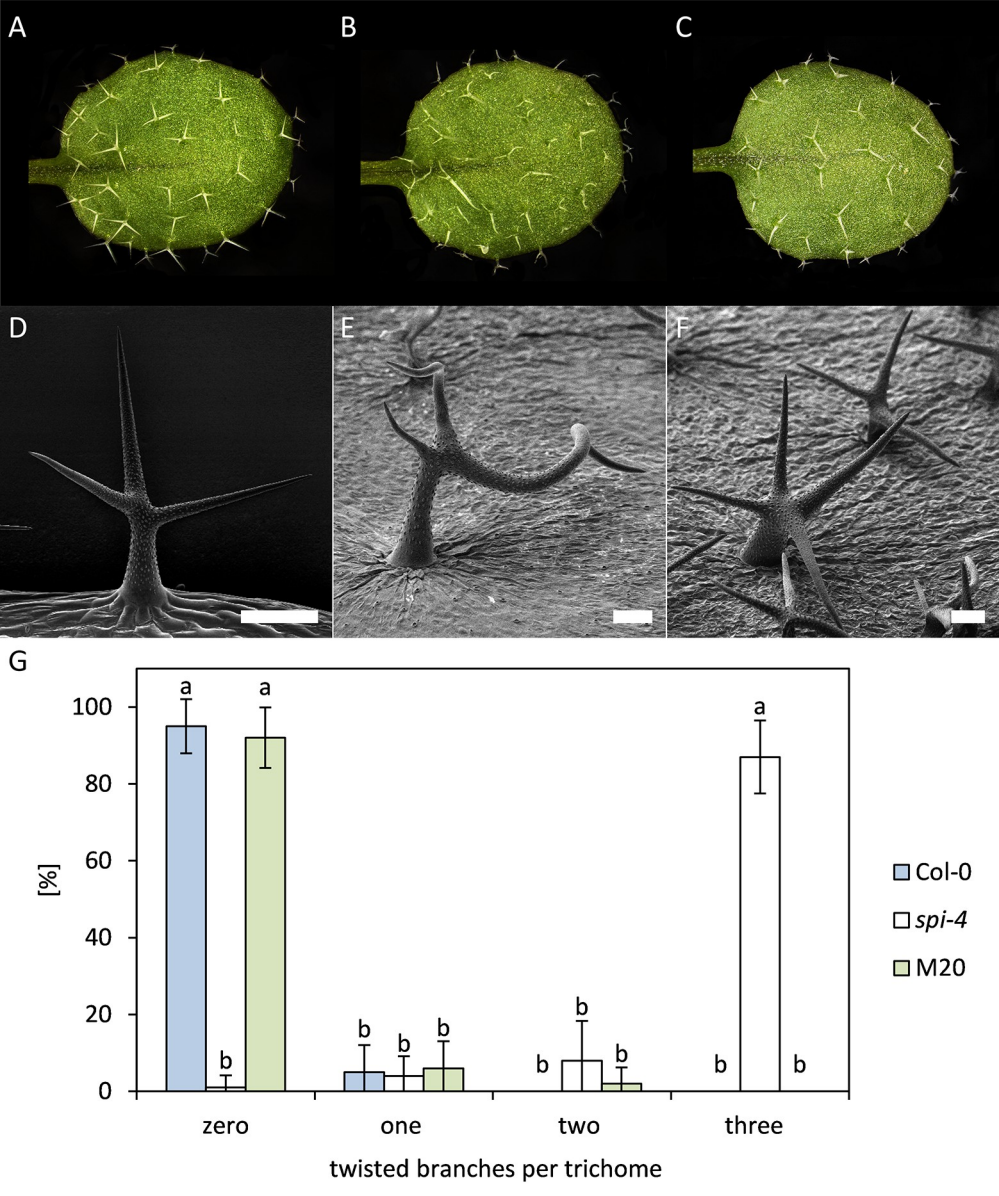
Rescue of the trichome phenotype. Representative pictures of rosette leaves of (A) Col-0, (B) *spi-4*, and (C) M20. Higher magnification REM pictures of (D) Col-0, (E) *spi-4*, and (F) M20. (G) Ten trichomes at the top half of ten leaves were analyzed for the number of twisted branches per trichome in Col-0, *spi-4*, and M20. The significance of the data was tested by a two-tailed T-test and is indicated by lowercase letters (p<0.001).

**Fig 2:**
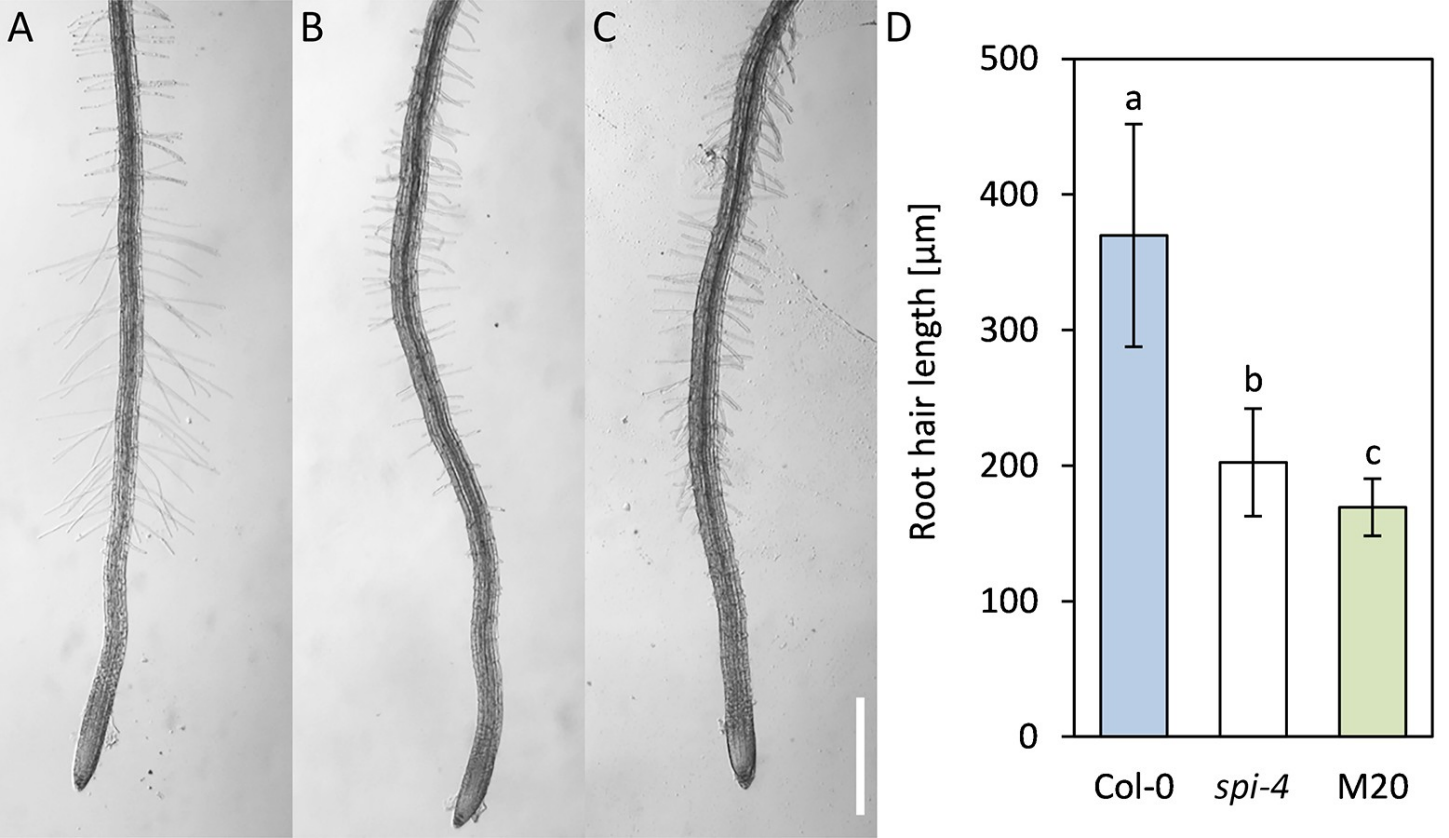
Root hair phenotype of Col-0, *spi-4*, and the M20 line. Light microscopy images of (A) Col-0, (B) *spi-4*, and (C) M20 roots grown on ½ MS agar plates. The scale bar displays 500 μm. (D) Barplot displaying root hair lengths of Col-0, *spi-4*, and M20. The significance of the data (n = 30 cells) was tested by a two-tailed T-test and is indicated by lowercase letters (p<0.001).

In *spi-4* mutants, trichome branches are curled and twisted (Fig 1B and 1E). We compared the percentage of curled/twisted trichomes in Col-0, *spi-4*, and the suppressor line M20. In *spi-4* mutants, 87% of the trichomes on true leaves show a curled/twisted phenotype of at least 3 trichome branches (Fig 1G). In Col-0 and the M20 line, the maximal number of twisted branches never exceeds two. In contrast, no twisted branches were found in 95% of Col-0 and 92% of M20 trichomes (Fig 1A, 1C, 2D, 1F and 1G), indicating that a second-site mutation rescues the *spi-4* mutant phenotype.

To assess whether the suppressor mutation is trichome-specific, we studied the rescue of several other *spi-4* mutant phenotypes. One cell morphogenesis phenotype of *spi-4* mutants is a reduced root hair length [5]. Plants were grown on ½ MS agar plates, and root hair length was determined on 5-day-old seedlings. Col-0 root hairs are, on average, 396.7±82.1 μm long, while *spi-4* mutant root hairs are 202.4±39.7 μm long (Fig 2A, 2B and 2D). The M20 line exhibited significantly shorter root hairs than *spi-4* mutants (Fig 2C and 2D; 169.1±21.0 μm), indicating that the root hair phenotype is not rescued in the suppressor line but rather enhanced.

**Fig 3:**
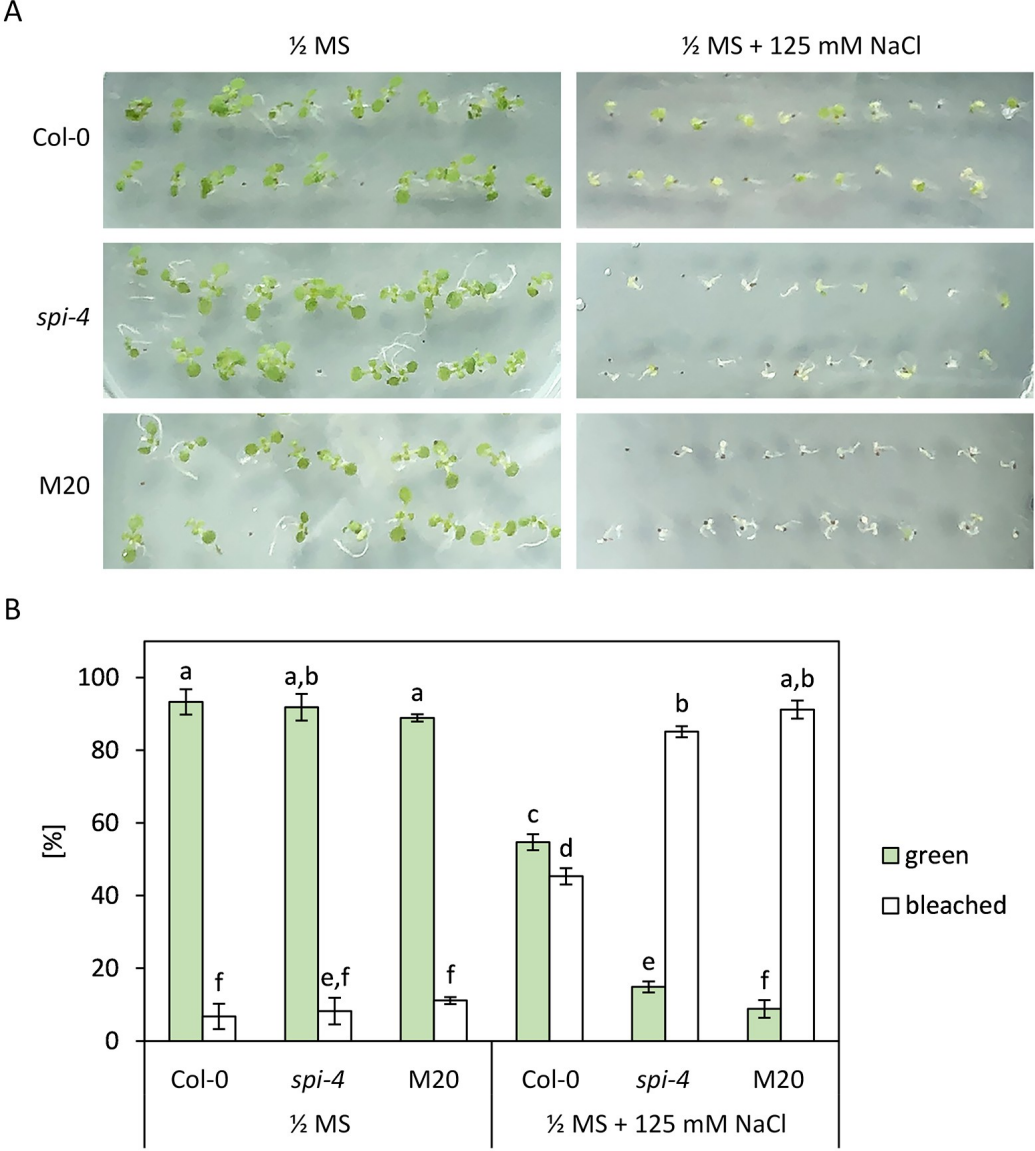
The effect of salt stress on Col-0, *spi-4*, and the suppressor line M20. Seeds of the respective lines were germinated on ½ MS medium. After visible germination, approx. 50 seeds were transferred to ½ MS plates (control) or ½ MS plates supplemented with 125 mM NaCl. Three independent technical replicates were analyzed, and green vs. bleached seedlings were counted after one week. (A) Representative pictures of treated plants; (B) Frequency of phenotypes. The significance of the data sets (n = 3 replicates) was tested by a Mann-Whitney-U test and is indicated by lowercase letters (p<0.1).

High concentrations of NaCl inhibit the growth of *spi-4* compared to Col-0 [8]. In three independent cotyledon greening assays (Fig 3), we investigated the effect of the suppressor mutation on salt resistance. Growth of wild-type Col-0 plants on 125 mM NaCl leads to bleaching of cotyledons in about 50% of the plants (Fig 3A and 3B). In *spi-4* mutants, more than 80% of the plants have white cotyledons under these conditions. The M20 line shows a similar phenotype as *spi-4*, indicating that the salt hypersensitivity phenotype of *spi-4* is not rescued (Fig 3A and 3B).

### 1.2 Identification of the *spi* suppressor mutation

**Fig 4:**
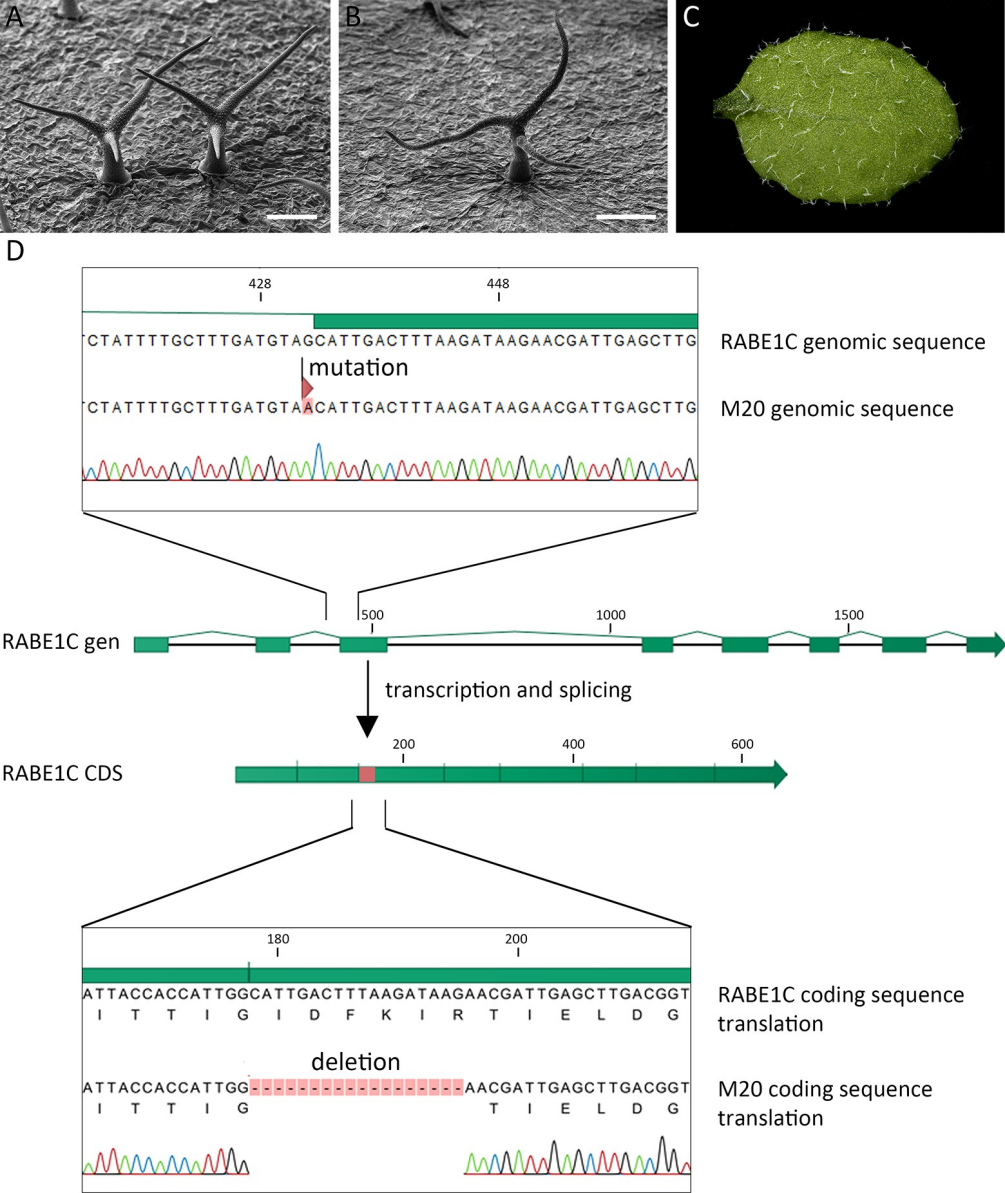
Complementation of the suppressor line M20. (A) REM picture of trichomes in the suppressor line M20 (*spi-4 rabe1c*). Expression of RABE1C from a 3.3 kb genomic fragment rescues the *rabe1c* mutation leading to the *spi-4* phenotype (cf. Fig 1): (B) REM picture of a trichome showing the restored *spi* phenotype. The scale bar in (A) and (B) displays 100 μm. (C) Overview of trichomes on a young rosette leaf showing the restored *spi* phenotype. (D) Schematic representation of the *RABE1C* gene (At3g46060) and transcript in the M20 line. M20 RABE1C exhibits a G>A mutation at the border of intron two to exon three, which leads to an 18 bp deletion in the coding sequence after splicing. Sequencing was carried out by GATC/Eurofins, Ebersberg.

To identify the *spi* suppressor mutation, we used a map-based cloning approach. The M20 line (*spi-4* in Col-0 background) was crossed with *spi-12* (L*er* background). Plants exhibiting trichome rescue in the F2 were mapped using CAPS and SSLP markers. Based on 24 plants, we localized the suppressor mutation to the lower arm of chromosome 3 (S1A Fig). For fine mapping, we included 90 additional plants. New SSLP markers were designed to cover the remaining interval on the lower arm of chromosome 3. This enabled us to map the gene to a region between At3g46630 and At3g46820 containing 153 genes (S1B Fig). We sequenced 15 candidate genes with predicted functions in vesicle trafficking or RNA metabolism (S1 Table). The *RABE1C* gene (At3g46060) exhibited a G>A mutation at the border of intron two to exon three, suggesting that this mutation leads to splicing defects. To verify that the mutation in *RABE1C* in the M20 line is responsible for rescuing the trichome phenotype of *spi-4*, a 3.3 kb genomic fragment of *RABE1C* was stably transformed into M20. In the T1, thirty-four separate transformants were identified that exhibited twisted trichomes and propagated to verify the phenotypic rescue. Thirty-one of these lines maintained the *spi* phenotype in the T2, indicating the second-site mutation was rescued (Fig 4A–4C).

#### Characterization of *RABE1C* in M20

Mutations in splice acceptor sites can have different consequences, including lower amounts of transcripts, alternative splice acceptor sites, or a premature stop codon. To test these possibilities, we amplified the cDNA of *RABE1C* from line M20 and sequenced the PCR product. We found an in-frame 18 bp deletion coding for amino acids 51–56 of RABE1C (RABE1C^Δ51–56^, Fig 4D). This region is named switch I and was shown to contact the *γ* phosphate of GTP [19].

#### The suppressor phenotype of M20 is mimicked by dominant negative RABE1C^S29N^

**Fig 5:**
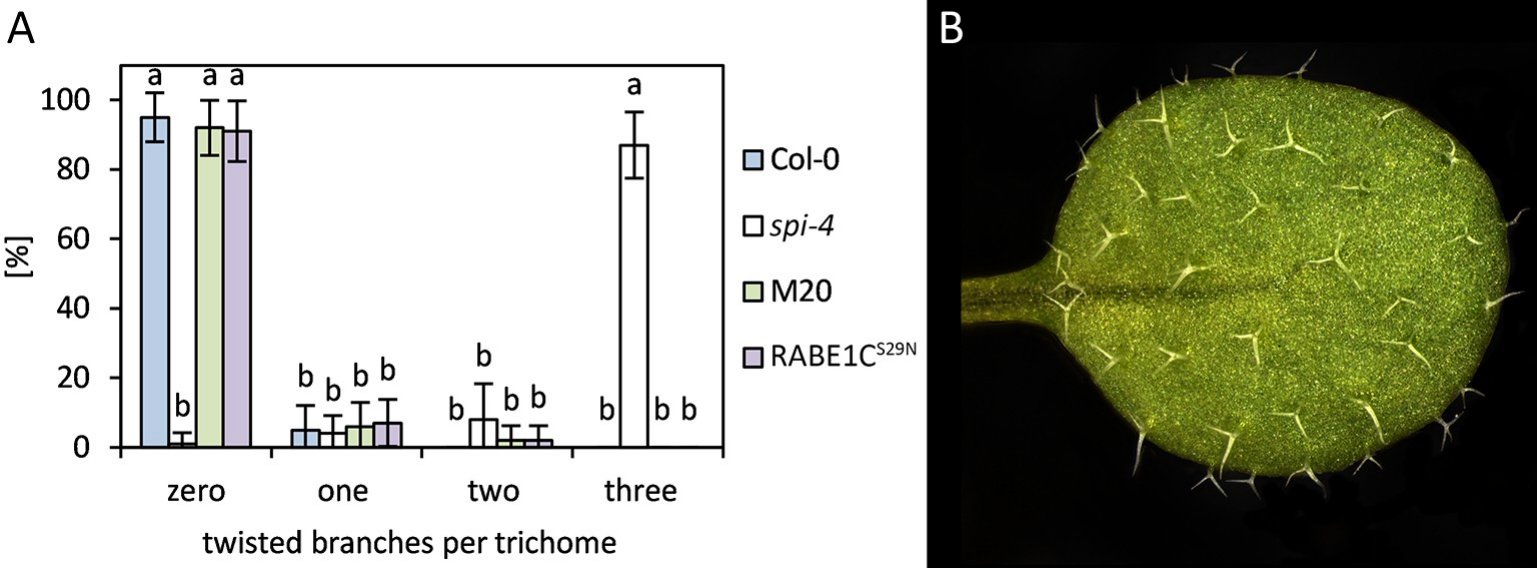
Rescue of the twisted trichome phenotype of *spi-4 with* RABE1C^S29N^. (A) Ten trichomes at the top half of ten leaves were analyzed for the number of twisted branches in *spi-4* RABE1C^S29N^. Data for Col-0, *spi-4*, and M20 from Fig 1G were added for comparison. The significance of the data was tested by a two-tailed T-test and is indicated by lowercase letters (p<0.001). (B) Rosette leaf of *spi-4* RABE1C^S29N^.

Deleting the switch I region in the suppressor RABE1C^Δ51–56^ is likely to result in an inactive protein variant. To test this, we generated an alternative inactive GDP-locked version of RABE1C by exchanging S for N at position 29. This *RABE1C*^*S29N*^ was introduced into *spi-4* as an N-terminal YFP fusion under the control of the UBQ10 promoter. Trichomes in the resulting eighteen T2 lines exhibited rescue of the twisted trichome phenotype of *spi-4* comparable to the mutation in the suppressor line M20 (Fig 5). This supports the idea that deleting amino acids 51–56 creates an inactive version of RABE1C.

#### RABE1C^Δ51–56^ shows decreased overlap with Golgi

RabE proteins are regulators of polarized vesicle transport from the Golgi apparatus to the plasma membrane. Recent research revealed that plant exocytosis is regulated through an interaction network involving RabE proteins, their GEFs, STOMATAL CYTOKINESIS DEFECTIVE 1/2 (SCD1/2) and exocyst proteins [20]. To investigate the effect of the amino acids 51–56 deletion on the localization of RABE1C, we performed co-localization experiments.

**Fig 6:**
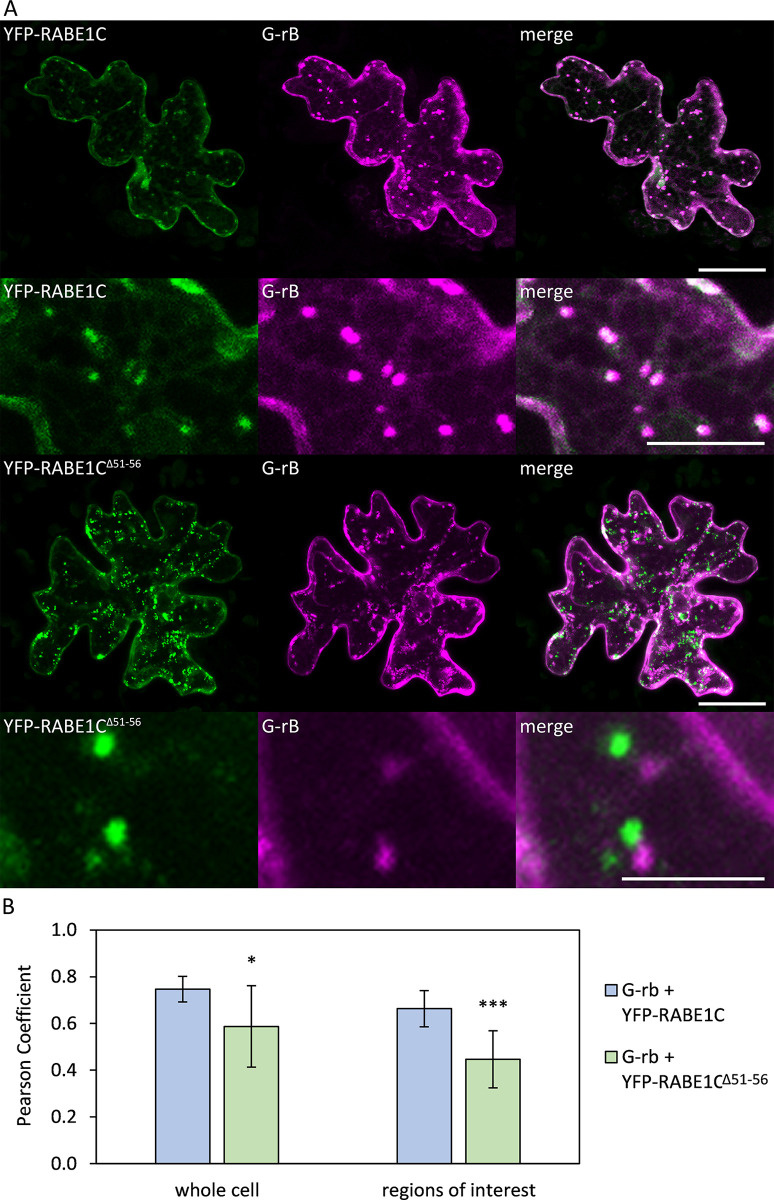
Co-localization of RABE1C and RABE1C^Δ51–56^ with the Golgi marker G-rb. (A) Transiently transformed *Arabidopsis thaliana* Col-0 cells. Top two rows from left to right: pUBI10:YFP-RABE1C, Golgi Marker G-rb, merged YFP and mCherry signals; bottom two rows from left to right: pUBI10:YFP- RABE1C^Δ51–56^, Golgi Marker G-rb, merged YFP and mCherry signals. The scale of the overview pictures (rows 1 and 3) displays 25 μm. The scales of the detailed pictures display 10 μm (row 2) and 5 μm (row 4). (B) Co-localization analysis. Co-localization was quantitatively analyzed using the Colocalization Threshold function in ImageJ. The Pearson coefficient r was determined for 10 cells. Depending on cell size, five to ten regions of interest were defined manually in areas that contained punctate structures and minimal diffuse fluorescence signals. One asterisk (*) indicates a significance level of p<0.05, three asterisks (***) indicate a significance level of p<0.001; significance was determined using a two-tailed T-test.

It was previously shown that RABE1D co-localizes with Golgi [21, 22]. Consistent with this, we found co-localization of RABE1C with the Golgi marker G-rb [23] in transiently transformed wild-type Col-0 epidermal cells (Fig 6A). Whole cells reached Pearson co-localization coefficients of r = 0.747, while regions with punctate structures reached r = 0.663 (Fig 6B). In contrast, co-localization of RABE1C^Δ51–56^ with Golgi was reduced to r = 0.587 for whole cells (Fig 6B; p = 0.016, two-tailed T-test), and punctate structures showed even less co-localization at r = 0.447 (Fig 6A, rows 3–4; Fig 6B, p = 1.99*10^−4^, two-tailed T-test).

**Fig 7:**
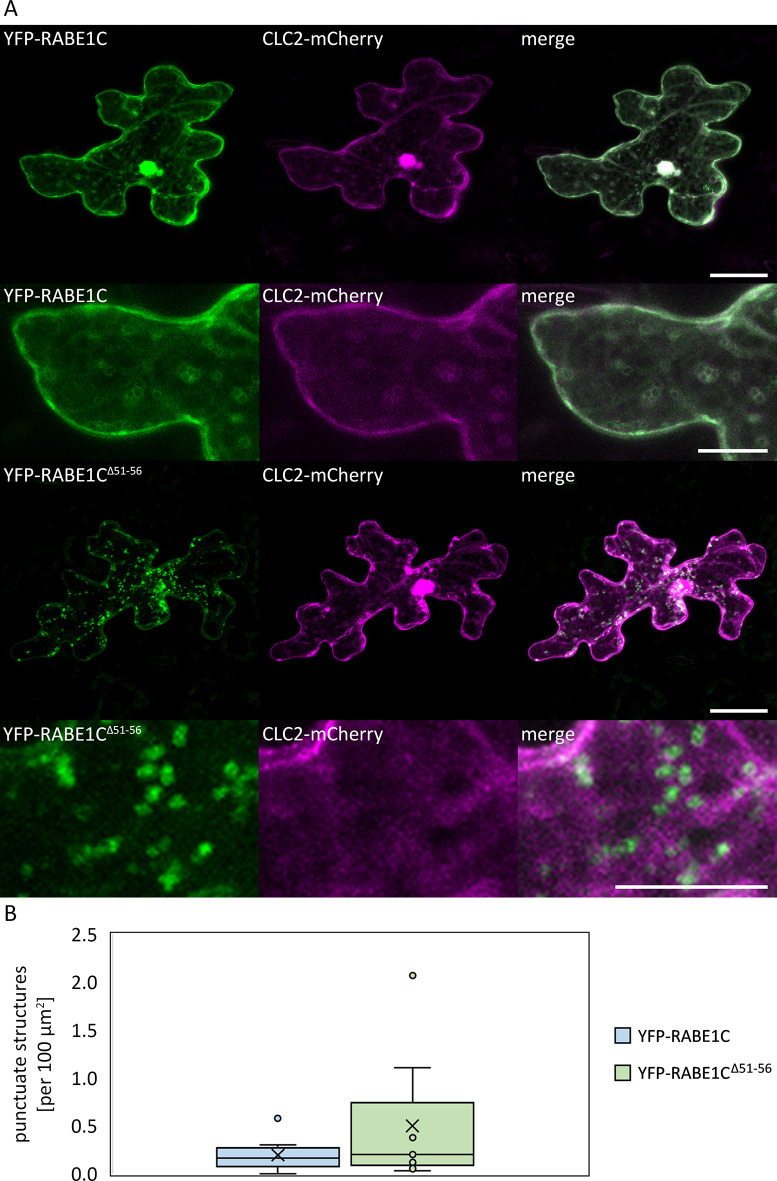
Localization of RABE1C and RABE1C^Δ51–56^ co-expressed with CLC2-mCHERRY. (A) Transiently transformed *Arabidopsis thaliana* Col-0 cells. Top two rows from left to right: pUBI10:YFP-RABE1C, CLC2:mCherry, merged YFP and mCherry signals; bottom two rows from left to right: pUBI10:YFP- RABE1C^Δ51–56^, CLC2:mCherry, merged YFP and mCherry signals. The scale of the overview pictures (rows 1 and 3) displays 25 μm. The scale of the detailed pictures displays 10 μm (row 2 and 4). (B) Boxplot comparing the density of punctate structures of RABE1C and RABE1C^Δ51–56^ when co-expressed with CLC2-mCherry. The means of the data are not significantly different in a two-tailed T-test at a significance level of p<0.05.

SCD1 and SCD2 proteins have been shown to associate with isolated clathrin-coated vesicles (CCV) and to co-localize with CLATHRIN LIGHT CHAIN 2 (CLC2) [24]. Therefore, we tested the co-localization of RABE1C with CLC2 (Fig 7A). Co-expression of RABE1C with CLC2 modified the localization of the Rab protein. We observed a more diffuse localization in the cytoplasm and surrounding vesicular structures. In contrast, RABE1C^Δ51–56^ remained predominantly in punctate structures. To quantify this mis-localization, we determined the number of punctate structures per area in both combinations, as a Pearson coefficient would have been unsuitable for depicting this structural difference. While dots were found at a mean density of only 0.195 per 100 μm^2^ for RABE1C, RABE1C^Δ51–56^ displayed a 2.57-fold increase to 0.501 dots per 100 μm^2^ (Fig 7B). These results are consistent with our previous data, as transport of RABE1C^Δ51–56^ diverges already at the Golgi and, most likely because of its inactive state, does not interact with SCD1/2 and the exocyst complex in later steps.

### 1.3 Excretion of proteins is altered in *spi* and partially rescued in M20

**Fig 8:**
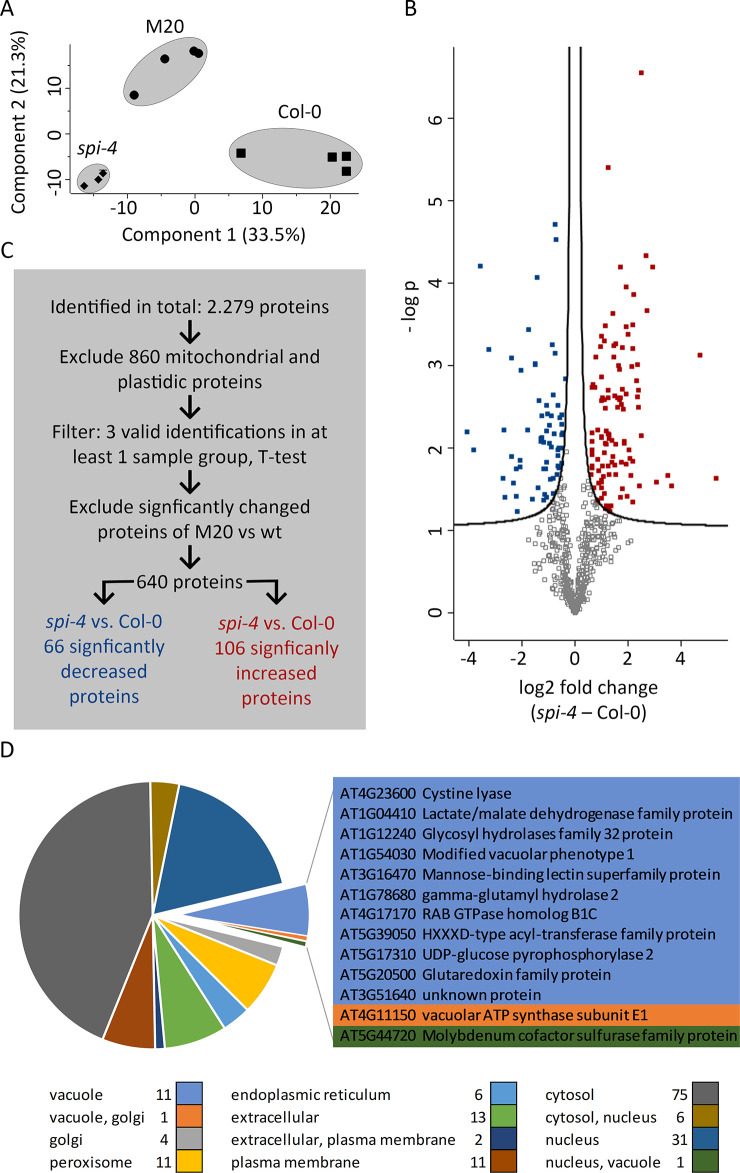
Proteomic analysis of excretion in *spi-4*, its suppressor line M20, and Col-0. (A) Principal component analysis of the proteomic results (n = 4). (B) Protein numbers and filter strategy for selecting candidate proteins showing significantly differential abundance in the excretions of *spi-4* and Col-0, but not between Col-0 and the suppressor line M20 (FDR: 0.05). (C) Volcano plot of the filtered data set, visualizing significantly increased (red) and decreased (blue) candidate proteins (FDR: 0.05). (D) Distribution of subcellular localization of differentially excreted proteins. All depictions are based on data from S2 Table.

It has been shown that a dominant negative form of Tobacco NtRab-E1^d^ inhibits transport to the plasma membrane [21]. In addition, by overexpression of RABE1D in *A*. *thaliana*, constitutive excretion of PATHOGENESIS-RELATED PROTEIN 1 (PR1) into the extracellular matrix was induced [22]. Conversely, we have shown that the normally vacuole-directed CARBOXYPEPTIDASE Y (CPY) is excreted in *spi* [6]. These findings prompted us to determine the composition of the excreted proteome in *spi-4* and its suppressor line M20. We generated mesophyll protoplasts of Col-0, *spi-4*, and M20. After 4 h of incubation, protoplasts were separated from the surrounding medium. The composition of proteins in the media samples was determined by LC-MS/MS, and subsequent data evaluation was performed as described in the method section (S2 Table and Fig 8). Under the assumption that secretion of specific proteins could be decreased in *spi-4* mutants but increased in the suppressor line or vice versa, we considered those proteins interesting, which were equally abundant in Col-0 and M20 but not in *spi-4*. We identified 640 proteins which were detected in at least 3 out of 4 samples for each line and did not show a significant difference between the suppressor line M20 and Col-0. Within this group, we found 66 proteins with significantly decreased abundance in *spi-4* and 106 proteins with significantly increased abundance (Fig 8A–8C).

Since we had previously shown that SPI is involved in cargo transport to the vacuole [6], the most promising candidates are within the 13 proteins with known vacuolar localization (Fig 8D). In addition, 15 extracellular proteins showing alterations in abundance between the lines (S2 Table and Fig 8) could also be interesting for further analysis.

## Discussion

BEACH domain proteins were initially thought to be mainly involved in cellular trafficking processes. Analysis of SPIRRIG broadened the view as it is involved in morphogenesis [5, 7] and transport to the vacuole [6], but also in actin organization in root hairs [4] and P-body activity [8]. Here, we conducted a suppressor screen to gain further insight into SPIRRIG´s function. We isolated a functionally inactive mutant of RABE1C, counteracting *spi* in trichome development. In this work, we have shown that an equivalent mutation in *RABE1C*, leading to an inactive GDP-locked version of RABE1C, can rescue some aspects of the *spi* mutant phenotypes. While the trichome phenotype is rescued, the root hair phenotype and the salt hypersensitivity phenotypes are not. This differential rescue indicates that one but not all SPI-dependent molecular pathways are affected by the second-site mutation.

A connection between excretory Rabs and BEACH domain proteins has been found before. RABE1C is a close homolog of Dictyostelium Rab8a [25], which is the target of the GTPase disgorgin. A null mutant of disgorgin is suppressed by a mutation in lvsA and enhanced by lvsD knockout; both genes encode for BEACH domain proteins closely related to SPIRRIG [25]. Recently, the direct involvement of Rab8 in secretion was shown by overexpression of GFP-Rab8 in Huh7 cells, which promoted exosome secretion [26]. Similarly, it was found that the Drosophila BDCP blue cheese (Bchs) acts as a functional antagonist of the excretory vesicle-trafficking regulator Rab11 [5].

One plausible explanation for the rescue of *spi* mutants by a second-site mutation in *RABE1C* is that a reduced transport to the vacuole in *spi* mutants is counteracted by a reduced transport to the plasma membrane due to the *rabe1c* mutation. This is consistent with our finding that RABE1C^Δ51-56^-labelled vesicles show reduced co-localization with Golgi, suggesting that a fraction of these vesicles does not follow the regular route to the plasma membrane. In addition, our proteomics data also identified several vacuolar proteins differentially excreted between *spi* on one side and Col-0 and M20 on the other.

We could envision two scenarios of how RABE1C^Δ51–56^ rescues the trichome phenotype. First, proteins needed for proper trichome development, which are normally transported to the vacuole, are excreted in *spi* mutants. In this scenario, M20 rescues by redirecting them to the vacuole. Second, proteins normally needed for proper trichome development are transported to the plasma membrane in excess, and the dominant negative version of RABE1C reduces this overflow.

Circumstantial evidence suggests that the second scenario is less likely. It has been previously shown that the constitutively active form of RABE1D (RABE1D^Q74L^) induces constitutive secretion of defense-related extracellular proteins [5]. However, while it is conceivable that the second-site mutation rescues the *spi* phenotype by re-routing relevant proteins in the post-Golgi pathways, it is currently not possible to pinpoint specific protein distribution changes explaining the rescue in M20.

## Material and methods

### Plant lines and growth conditions

*Arabidopsis thaliana spi-4* was described before [8]. Transgenic lines were generated by floral dipping [27]. Plants were grown on soil or surface-sterilized and grown on ½ MS plates [28]. For surface sterilization, a spatula tip of seeds was added to a microcentrifuge tube and positioned in a desiccator. A beaker containing 100 ml NaOCl was placed into the desiccator, and 5 ml of 37% HCl were added. The seeds were sterilized with the developing chlorine gas for at least 4 h. Seeds were stratified for at least three days and subsequently transferred to long-day conditions at 21±1°C, 60% humidity, and 100±20 μmol/m^2^s light intensity.

### EMS mutagenesis

EMS mutagenesis was carried out as described in [29]. Seeds from M2 families were sown in seed trays on soil and screened for the reversion of the *spi* phenotype under a stereo microscope.

### Root-hair length measurements

For root-hair length measurements, plates were placed upright, and pictures were taken after 4 days under a DM5000 B microscope at 10x magnification (Leica, Germany). Images were analyzed using ImageJ (Fabrice Cordelieres, Institut Curie, Orsay, France).

### Salt treatment of plants

Seeds of the respective lines were germinated on ½ MS medium. After visible germination, approx. 50 seeds were transferred to ½ MS plates (control) or ½ MS plates supplemented with 125 mM NaCl. Three independent technical replicates were analyzed, and green vs. bleached seedlings were counted after one week.

### Sequence analysis and plasmids

Sequences were taken from TAIR [30] and NCBI (National Centre for Biotechnology Information, www.ncbi.nlm.nih.gov). *In silico* sequence analysis was carried out with CLC DNA Workbench version 5.6.1.

The construct for the complementation of M20 was created by amplifying a 3.3 kb fragment comprising the genomic region of RABE1C with primers J1526 and J1527. The resulting fragment was digested with AscI and XbaI and ligated to the backbone of pENSG-YFP digested with the same enzymes. The coding sequences of RABE1C and RABE1C^Δ51–56^ were amplified with primers J1529/J1530 from Col-0 and M20 cDNA, respectively. The resulting fragments were cloned in pDONR207 (Invitrogen) and sequenced. RABE1C^S29N^ was created via site-directed mutagenesis of pDONR207-RABE1C. All RABE1C derivatives were transferred to pENSG-YFPpUBI for expression under the ubiquitous UBQ10 promoter. G-rb was received from ABRC [23]. The CLC2-mCherry marker was created by amplifying the coding sequence of CLC2 with primers J1655/1656 and cloning in pDONR207. After sequencing, CLC2 was transferred to pAUBERGINEpUBI for C-terminal fusion to mCherry and expression under the UBQ10 promoter. A list of primers is displayed in S3 Table. All constructs used in this study were confirmed by sequencing (GATC/Eurofins, Ebersberg).

### Plant expression of fusion proteins

Transient expression of plasmids was carried out in rosette leaves of two-week-old Arabidopsis seedlings by biolistic transformation [31] and analyzed by confocal laser scanning microscopy after 12 to 16 h.

### Microscopy

Confocal laser scanning microscopy was carried out with the Leica TCS-SP8 (HC PL APO CS2 20x0.75 water-immersion objective) imaging system (Leica Microsystems, Heidelberg, Germany). YFP was excited at 514 nm, and emission was detected between 530 and 570 nm. mCherry was excited at 561 nm, and emission was detected between 600 and 635 nm. Sequential scanning between frames was used to avoid cross-talk between different fluorescently-tagged proteins.

### Co-localization analysis

Co-localization was quantitatively analyzed from confocal images using the Colocalization Threshold function of ImageJ. The Pearson coefficient r was determined for whole cells and five to ten regions of interest, which were defined manually in areas containing punctate structures and minimal diffuse fluorescence signals.

The number of punctate structures was manually counted and correlated to the area of the cells, which was determined using the polygon tool in ImageJ.

### Excretome of Col-0, *spi-4*, and M20

Mesophyll protoplasts of Col-0, *spi-4*, and M20 were generated (S2 Fig) and kept in 4 ml of MMg solution [32]. Four replicates were produced for each line. After 3 hours, protoplasts were separated from the surrounding medium. The supernatants (3 ml) were loaded on VIVASPIN 6 (10 kDa) columns and washed two times with 3 ml of 4 mM MES buffer (pH 5.7). The remaining 200 μl were precipitated with 800 μl Acetone overnight at -20°C. The pellets were dissolved in 8 M Urea, 20 μl Proteinase inhibitor, and 5 mM DTT. Sample treatment and data evaluation were essentially performed as described in Wolff et al. [33]. In short, after tryptic digestion, the samples were loaded onto StageTips to deplete salts and other contaminants prior to LC-MS/MS analysis. The protocols, solutions, chemicals, and styrene-divinylbenzene–reversed phase sulfonate discs-containing C18 StageTips were provided by the Proteomics Core Facility Cologne (http://proteomics.cecad-labs.uni-koeln.de). Subsequent LC-MS/MS analysis was carried out by the Proteomics Core Facility Cologne utilizing an EASY nLC 1200 UPLC (Thermo Scientific) and a Q-Exactive Plus (Thermo Scientific) mass spectrometer. The raw data of the MS2 spectra was processed by S. Müller, Proteomics Core Facility Cologne, using the Maxquant software (version 1.5.2.8.) set to default parameters. As a reference, the Uniprot ARATH.fasta database (download 16.06.2017) was used, which included common contaminants. The protein and peptide spectrum matches (PSM) false discovery rates (FDRs) were estimated using the target-decoy approach (1% Protein FDR and 1% PSM FDR). Only peptides with a minimum length of at least 7 amino acids were included in the analysis, and the carbamidomethylation of cysteines was set as a fixed modification. Oxidation and acetylation as variable modifications were included in the analysis. The match between runs option was enabled and used to raise the number of identified proteins. Default settings were employed for the calculation of intensity-based absolute quantification (iBAQ) and label-free quantification (LFQ) values. LFQ values allow a quantitative comparison of individual proteins across samples. We used the Perseus software (Version 1.6.1.1) [34] to further process the data, based on the LFQ values, to identify proteins with altered abundances in the excretions of the lines. In short, the MaxQuant output (2349 proteins) was filtered to remove non-plant contaminants, reversed sequences, and proteins that were only identified based on modified peptides. An in-house database, based on TAIR10 (https://www.arabidopsis.org/), mapman.org [35], and suba.live [36] was used to annotate the proteins with names and subcellular localization. Mitochondrial and plastidic proteins are typically not excreted but present in the analyzed excretomes due to damaged protoplasts. They were excluded from the data set. The LFQ values of the remaining 1419 proteins were then log2-transformed, and proteins were excluded from further analysis if they were not detected in at least three of the four biological replicates in at least one sample group (*spi-4*, M20, or Col-0). Remaining missing LFQ values were replaced by low, random values from a normal distribution. The samples were statistically evaluated by performing a volcano plot (T-test:FDR: 0.05, s0: 0.1) with the Perseus software. Proteins that showed a significant difference between the suppressor line M20 and Col-0 were excluded. A final volcano plot (T-test: FDR: 0.05, s0: 0.1) of the remaining 640 proteins for *spi-4* mutant vs. Col-0 was constructed, and lists of significantly increased and decreased proteins were extracted (S2 Table). Additionally, iBAQ values are provided in S2 Table to give information about the absolute quantity of individual proteins in the excretomes. They were initially calculated by the MaxQuant software. In short, summed intensities of all identified peptides of an individual protein were divided by the theoretically observable number of tryptic peptides. To simplify the data, we calculated relative iBAQs (riBAQs in %) by dividing each individual iBAQ by the total iBAQ of the sample, multiplied by 100.

## Supporting information

S1 Fig Markers tested in the first round of mapping the suppressor. (A) Chromosomal positions of the markers and their segregation for the Col-0 vs. the L*er* allele is shown in the format “marker_segregation Col-0:L*er*”next to the chromosomes depicted in light gray (cf. S1 Table). (B) Fine mapping of the chromosomal region of the suppressor. In the pool of 90 plants, 9 were identified that had recombinations on the lower arm of chromosome 3. Abbreviations are C = Col-0, L = L*er*, CL = heterozygous. The region between At3g46614 and At3g47180 (green) was used for further analysis.
(PDF)

S2 Fig SDS gel of different samples taken during the protoplasting process. (1) Protoplasting solution containing cellulase and macerozyme; (2) Page Ruler prestained protein marker (Fermentas); (3)-(6) Samples taken from the medium of Col-0 protoplasts after 1–4 h of incubation, respectively; (7)-(10) Samples taken from the medium of *spi-4* protoplasts after 1–4 h of incubation.
(PDF)

S1 Table Candidate genes for a *spi* suppressor. Genes in the mapped region related to endosomal trafficking and RNA metabolism selected for sequencing.
(XLSX)

S2 Table Excretome results. (XLSX)

S3 Table Primer list. (XLSX)

S1 Raw image (PDF)
