# Supplementary material for: Mutations in RABE1C suppress the spirrig mutant phenotype
Source: PLoS One. 2024 Jun 17;19(6):e0304001. doi: 10.1371/journal.pone.0304001 (PMC11182498; doi:10.1371/journal.pone.0304001)
Supplement: S1 Fig — (A) Chromosomal positions of the markers and their segregation for the Col-0 vs. the Ler allele is shown in the format “marker_segregation Col-0:Ler”next to the chromosomes depicted in light gray (cf. S1 Table). (B) Fine mapping of the chromosomal region of the suppressor. In the pool of 90 plants, 9 were identified that had recombinations on the lower arm of chromosome 3. Abbreviations are C = Col-0, L = Ler, CL = heterozygous. The region between At3g46614 and At3g47180 (green) was used for further analysis. (PDF) [file pone.0304001.s001.pdf]

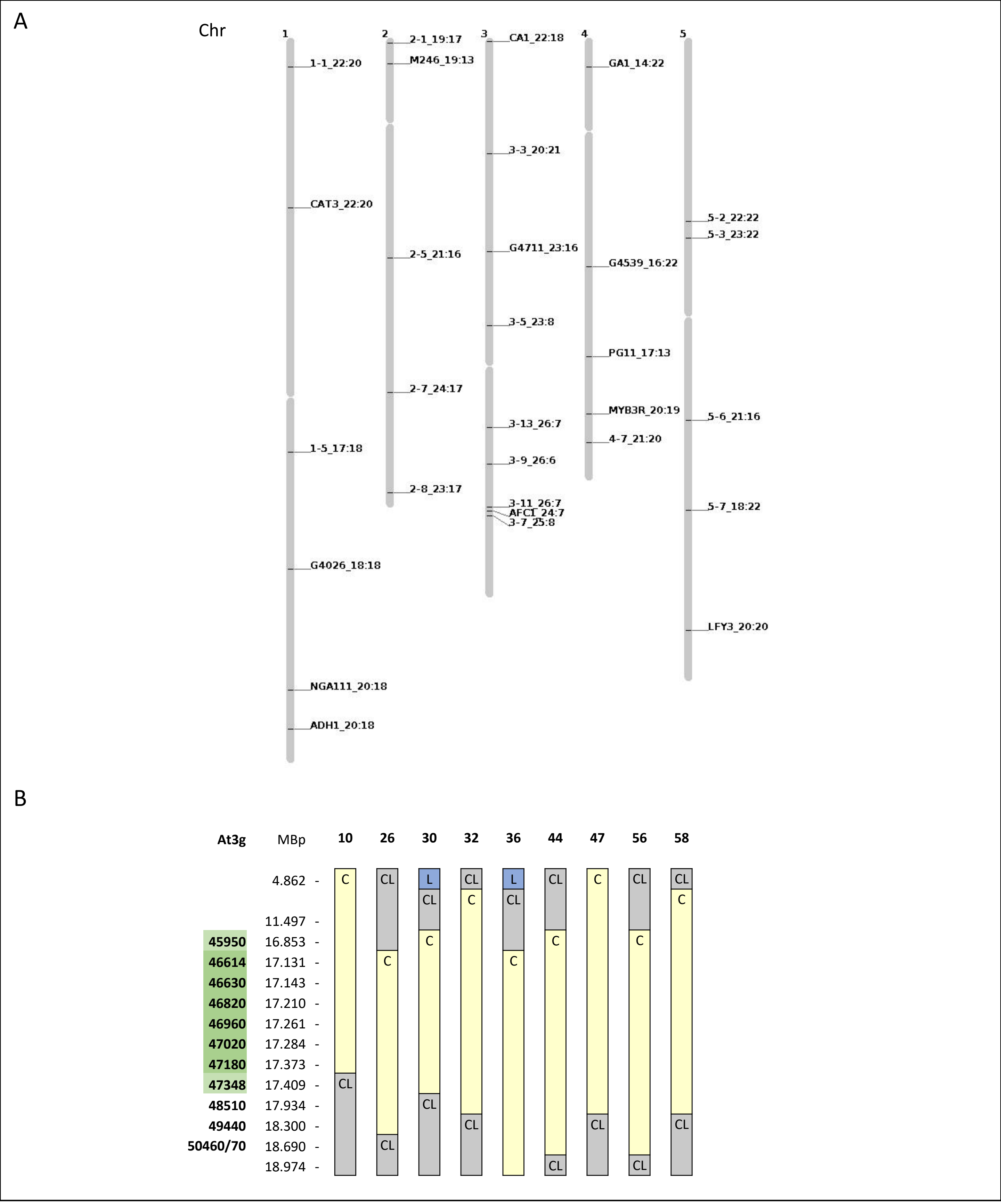

S1 Fig. Markers tested in the first round of mapping the suppressor.

(A) Chromosomal positions of the markers and their seggregation for the Col-0 vs. the L*er* allele is shown in the format “marker_seggregation Col-0:L*er*“ next to the chromosomes depicted in light gray (cf. S1 Table). (B) Fine mapping of the chromosomal region of the suppressor. In the pool of 90 plants, 9 were identified that had recombinations on the lower arm of chromosome 3. Abbreviations are C = Col-0, L = L*er* , CL = heterozygous. The region between At3g46614 and At3g47180 (green) was used for further analysis.
