# Supplementary material for: Mutations in RABE1C suppress the spirrig mutant phenotype
Source: PLoS One. 2024 Jun 17;19(6):e0304001. doi: 10.1371/journal.pone.0304001 (PMC11182498; doi:10.1371/journal.pone.0304001)
Supplement: S1 Raw image — (PDF) [file pone.0304001.s006.pdf]

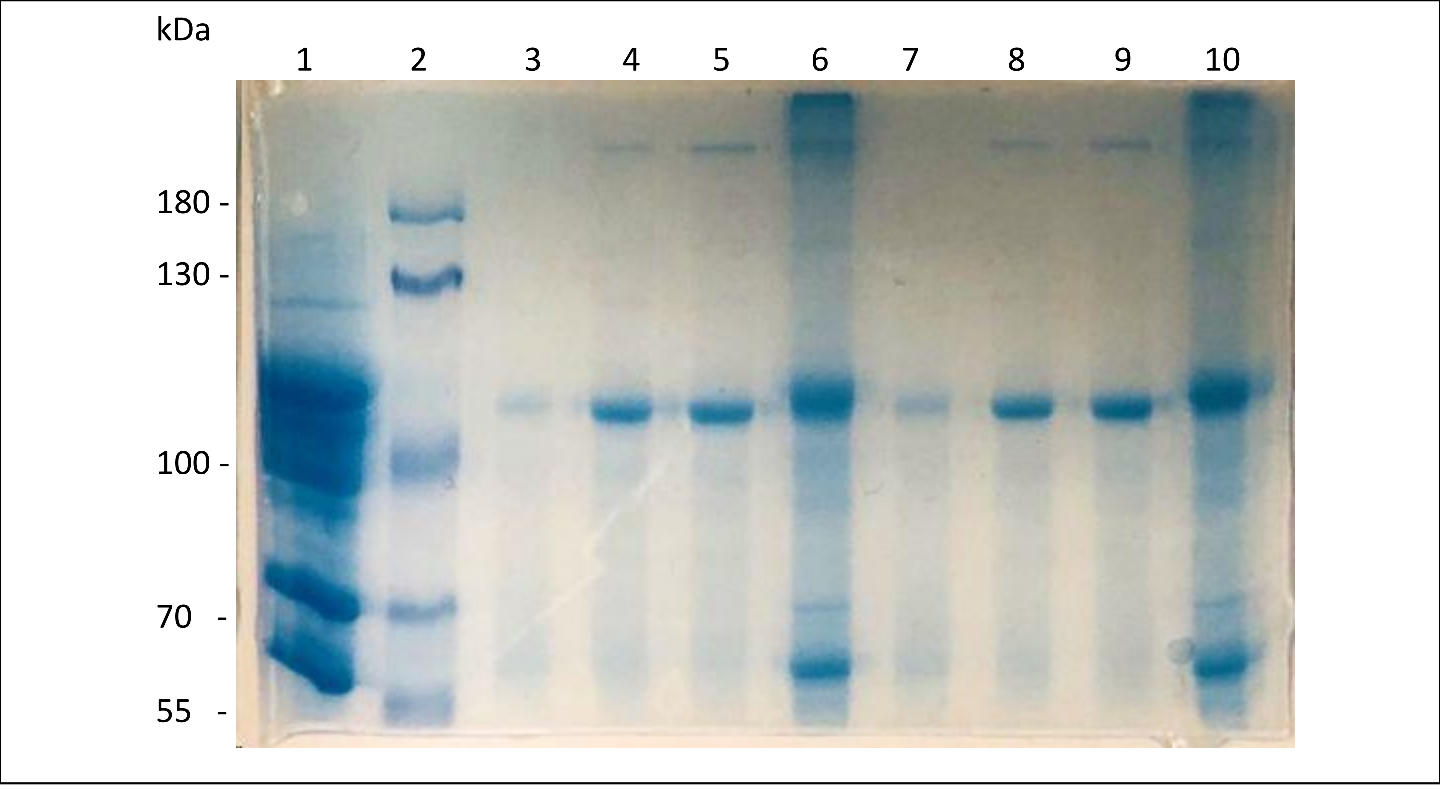

Raw Image of S2 Fig. SDS gel of different samples taken during the protoplasting process.
(1) Protoplasting solution containing cellulase and macerozyme; (2) Page Ruler prestained protein marker (Fermentas); (3)-(6) Samples taken from the medium of Col-0 protoplasts after 1-4 h of incubation, respectively; (7)-(10) Samples taken from the medium of *spi-4* protoplasts after 1-4 h of incubation. The picture was taken with a mobile phone and cropped to the margins of the gel upon recording.
